# The Expression of Water and Ion Channels in Diffuse Alveolar Damage Is Not Dependent on DAD Etiology

**DOI:** 10.1371/journal.pone.0166184

**Published:** 2016-11-11

**Authors:** Ruy Camargo Pires-Neto, Fabiola Del Carlo Bernardi, Priscila Alves de Araujo, Thais Mauad, Marisa Dolhnikoff

**Affiliations:** Departamento de Patologia da Faculdade de Medicina da Universidade de São Paulo, São Paulo, SP, Brazil; Institute of Biomedicine of Seville-IBiS, SPAIN

## Abstract

**Introduction:**

Aquaporins and ion channels are membrane proteins that facilitate the rapid movement of water and solutes across biological membranes. Experimental and in vitro studies reported that the function of these channels and pulmonary edema resolution are impaired in acute lung injury (ALI). Although current evidence indicates that alveolar fluid clearance is impaired in patients with ALI/diffuse alveolar damage (DAD), few human studies have addressed the alterations in pulmonary channels in this clinical condition. Additionally, it is not known whether the primary cause of DAD is a relevant variable for the channel dysfunction.

**Methods:**

Autopsied lungs of 43 patients with acute respiratory failure (ARF) due to DAD of three different etiologies, non-pulmonary sepsis, H1N1 viral infection and leptospirosis, were compared to 18 normal lungs. We quantified the expression of aquaporin (AQP) 1, AQP3, AQP5, epithelial Na^+^ channel (ENaC) and sodium potassium ATPase (Na-K-ATPase) in the alveolar septum using immunohistochemistry and image analysis.

**Results:**

The DAD group presented with increased expression of AQP3, AQP5 and Na-K-ATPase and decreased expression of ENaC compared to controls. However, there was no difference in protein expression within the DAD groups of different etiologies.

**Conclusion:**

Water and ion channels are altered in patients with ARF due to DAD. The cause of DAD does not seem to influence the level of impairment of these channels.

## Introduction

Under normal conditions, except for a small amount of fluid that lines the alveolar surface, alveolar spaces are kept dry for gas exchange to occur without a fluid barrier. In the case of an alveolar edema, the removal of fluid from the alveolar space is driven by active ion pumps and water channels located mainly in the apical and basolateral surface of pneumocytes (P) I and II [1].

Aquaporins (AQP) are integral membrane proteins that function as molecular water channels in a variety of fluid transporting tissues, including the lung [1]. To date, 12 subtypes of AQPs have been described, three of which are expressed in the lungs with a role in alveolar fluid clearance. AQP1 was the first identified aquaporin and is expressed in alveolar endothelial cells [2]. During the perinatal period, the increased expression of AQP1 parallels increased lung water permeability [2]. AQP3 is expressed by PII type cells, facilitating water and glycerol transport across the plasma membrane [3]. Although the exact mechanism is not well understood, AQP3 expression is increased in human lung carcinomas, particularly adenocarcinomas [4]. AQP5 is located in the apical surface of PI and is responsible for the majority of water transport across the apical membrane of this cell type [5].

In addition to the fluid transport associated to AQPs, under normal conditions the transepithelial fluid movement in the alveolar region is mainly driven by active salt transport [5, 6]. The epithelial sodium channel (ENaC) is located in the apical region of PI and PII. ENaC knockout mice present with persistent lung fluid at birth and die from respiratory failure. The sodium potassium ATPase (Na-K-ATPase) is located in the basolateral surface of PI and PII. In addition to ion transport, it is involved in the formation and maintenance of intercellular junctions. Alveolar fluid clearance (AFC) can partially be explained by sodium conductance. ENaC pumps Na^+^ from the alveolar space into the cell, whereas Na-K-ATPase pumps Na^+^ out from the cell, thereby regulating cell volume and glucose and amino acid transport [5, 7].

Lung injury in diffuse alveolar damage (DAD)/acute respiratory distress syndrome (ARDS) is characterized by alveolar flooding due to capillary alveolar membrane inflammatory lesions. In this case, the reabsorption of pulmonary edema is essential for the resolution of the injury [5, 8]. In fact, it has been shown in ARDS patients in whom AFC is impaired that a better AFC is associated with improved outcomes with lower mortality [9]. Previous studies in animal and cell culture models reported that water and ion channels are impaired after an experimental acute lung injury (ALI) [4, 10–13]. However, there is currently little data on the alterations of these channels in human lungs. Additionally, different models of ALI have yielded conflicting results [11, 12]. Therefore, it is not clear whether the etiology of the injury is related to the altered expression and function of the channels [11].

DAD is related to different pulmonary insults that may present with distinct pathophysiologies. Sepsis is the major cause of extrapulmonary ARDS and leads to DAD primarily through capillary injury [8]. Pulmonary viral infection and leptospirosis cause direct injury to the alveolar-capillary membrane and, in their most severe forms, are known risk factors for ARDS. Although DAD in these conditions is uncommon, it is associated with a high rate of mortality [14, 15]. We hypothesized that water and ion pump channels are distinctly impaired in DAD of different etiologies. Therefore, the objective of this study was to evaluate the expression of water and ion pump channels in the lungs of patients with acute respiratory failure due to DAD and compare them to control subjects that presented with a non-pulmonary cause of death. The samples were also compared for the expression of the different water and ion channels according to DAD etiology.

## Methods

This study was approved by the review board for human studies of the São Paulo University Medical School (Comissão para Análise de Projetos de Pesquisa—CAPPesq-FMUSP n° 355/10). The study is retrospective and used archived material from routine autopsies performed at the Autopsy Service of Sao Paulo University Medical School.

### Study population

Lung tissues from 71 patients submitted for autopsy at Sao Paulo University Medical School were retrospectively included in this study and divided into two groups. The DAD group (n = 43) was characterized by patients with acute respiratory failure of different etiologies, presenting with histological findings of DAD [16] and the absence of chronic lung diseases. Three different etiologies of DAD were considered in this group as follows: 1) non-pulmonary sepsis; 2) H1N1 viral infection and; 3) leptospirosis. The diagnosis of non-pulmonary sepsis was clinically assessed according to the International Guidelines for Management of Severe Sepsis by the presence of a non-pulmonary infection and clinical systemic manifestations [17]. The H1N1 group was characterized by pulmonary injury due to an A(H1N1)pdm09 viral infection. The diagnosis was confirmed in nasopharyngeal swab specimens using a real-time reverse transcriptase polymerase chain reaction (rRT-PCR) test, in accordance with the guidelines from the Centers for Disease Control and Prevention (CDC) [15]. Leptospirosis diagnosis was clinically assessed and confirmed either by an at least 4-fold increase in the microagglutination titer, a single microagglutination titer greater than 1:400, and/or detection of leptospiral antigen in the biopsy or autopsy specimens [14, 18]. We described these cohorts in previous studies [15, 18–20].

The control group (n = 28) included non-smoker, non-ventilated patients without previous lung diseases who died of non-pulmonary causes. Some of these patients presented areas of alveolar edema due to heart failure, but all patients showed normal lung architecture upon gross and microscopic examination.

### Tissue Processing and Histological Analysis

Paraffin blocks of lung tissue collected during routine autopsy were retrieved from the archives of the Department of Pathology of Sao Paulo University Medical School. Three to four fragments of lung tissue were collected from regions of altered lung parenchyma. In normal lungs, one fragment of lung tissue was collected from each lobe. The tissue was previously fixed in 10% buffered formalin for 24 hours, routinely processed and paraffin embedded. Five μm-thick sections were stained with hematoxylin and eosin (H&E) for histological diagnoses of DAD. Two to three slides per patient containing alveolar septum were selected for analysis and immunohistochemical staining.

The following proteins were identified with immunohistochemistry as previously described [19, 20]: aquaporin 1 (AQP1), aquaporin 3 (AQP3), aquaporin 5 (AQP5), sodium potassium ATPase (Na-K-ATPase) and epithelial sodium channel (ENaC). The antibody types and pre-treatment used are shown in Table 1.

**Table 1:** Antibodies and processing used in immunohistochemical analyses.

Antibody	Origin	Pre-treatment	Specie	Clone	Dilution	Secondary Antibody
Aquaporin 1	Sigma (St. Louis, MO/USA)	Citrate	Rabbit	Polyclonal	1:200	Envision (Dako—Glostrup, Denmark)
Aquaporin 3	Abcam (Cambridge, MA/USA)	Citrate	Rabbit	Polyclonal	1:1500	Novolink (Leica—Wetzlar, Germany)
Aquaporin 5	Abcam (Cambridge, MA/USA)	Citrate	Rabbit	EPR3747	1:400	Novolink (Leica—Wetzlar, Germany)
Na-K-ATPase_(α)_	Abcam (Cambridge, MA/USA)	Citrate	Mouse	464.6	1:800	Novolink (Leica—Wetzlar, Germany)
ENaC_(δ)_	Abcam (Cambridge, MA/USA)	Citrate	Rabbit	Polyclonal	1:2500	Envision (Dako—Glostrup, Denmark)

ENaC: Epithelial Na^+^ Channel

In order to describe the immunolocalization of aquaporins and ion channels within the alveolar tissue, a double staining with TTF-1 (Novocastra, Newcastle, UK, SPT 24 mouse anti- human, 1:100), a marker of PII cell, was performed in two slides for each channel staining.

After immunohistochemistry staining, each slide was scanned using a Panoramic Viewer^®^ 1.15.2 for Windows^®^ software (3DHistech, Budapest, Hungary). Protein expression was assessed using Image-Pro Plus^®^ 6.0 for Windows^®^ image-analysis software (Media Cybernetics, Silver Spring, MD, USA), as follows: At least 20–30 fields per case were selected for image analysis, with each field containing 1000 μm of alveolar septum at a 500x magnification. In each field, the area of alveolar septa was manually demarcated with the analyzer drawing tool. After the color threshold setting (see below), the area of positive staining within the septum was automatically calculated. The corresponding length of alveolar septum was also measured manually. The expression of the AQP1, AQP3, AQP5, ENaC, and Na-K-ATPase proteins in the alveolar septum was calculated as the area of positive staining normalized by the corresponding alveolar septum length (μm^2^/μm). The positive staining areas were determined by color threshold. For this purpose, different sections stained with each antibody, as well as negative controls, were used to achieve the best range of positivity for each case. This procedure generated a file containing all color selection data that were then applied to all fields stained with the same antibody [17, 20]. All slides were previously coded and analyzed by an investigator blinded to the groups.

### Statistical Analysis

Statistical analysis was performed using the statistical software GraphPad Prism version 5.00 for Windows (GraphPad Software, San Diego, California USA). A Mann-Whitney test or unpaired “t” test was used to compare data between the DAD and control groups according to the data distribution. Furthermore, we compared the protein expression within the DAD group according to the DAD etiology (leptospirosis, H1N1 and sepsis) using a Kruskall-Wallis test followed by Dunn’s post-test or one way ANOVA followed by Bonferroni’s post-test.

Data are presented as the mean ± SD or median [IQR]. The level of significance was set at p≤0.05.

## Results

### Study Population

**Fig 1:**
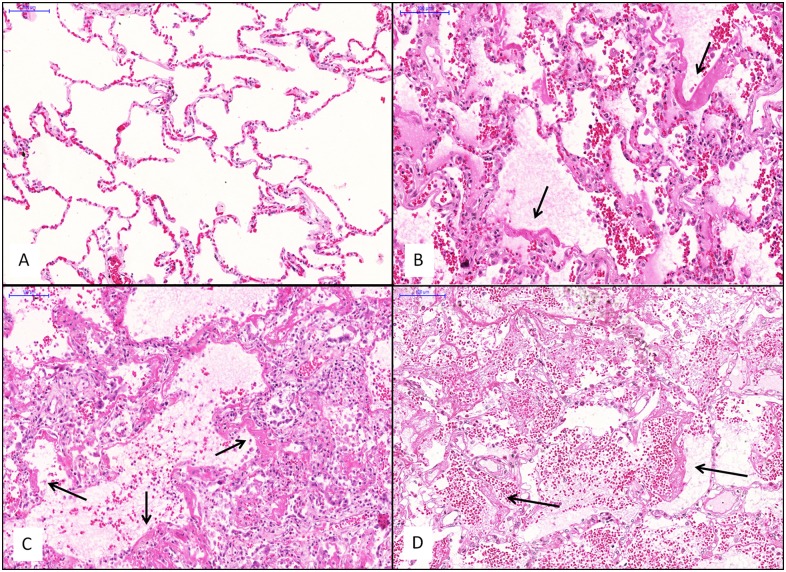
Representative histological images of each group. H&E staining. A: control group; B: DAD group (sepsis); C: DAD group (H1N1); D: DAD group (leptospirosis). Note the hemorrhage present in the leptospirosis group. Arrows: Hyaline membrane. Bars = 100 μm.

The demographic and clinical data for the DAD and control groups are presented in Table 2. Within the DAD group, 14 patients died of sepsis, 15 patients presented with H1N1 infection and 14 patients had leptospirosis. Patients with sepsis showed the classical histological presentation of DAD, characterized by hyaline membranes, intraalveolar edema, and neutrophilic exudates. Patients with H1N1 infection showed extensive diffuse alveolar damage with variable degrees of pulmonary hemorrhage and necrotizing bronchiolitis. DAD in the leptospirosis patients was characterized by extensive alveolar hemorrhage, mild inflammation and focal fibrin deposition/hyaline membranes. Representative histological pictures are shown in Fig 1.

**Table 2:** Demographic and Clinical Data.

Characteristics	Control (n = 28)	DAD (n = 43)		
		Sepsis (n = 14)	H1N1 (n = 15)	Lepto (n = 14)
**Age (years)**	54 ± 15	43 ±16	50 ± 13	41 ± 19
**Sex (M/F)**	15/13	6/8	9/6	13/1
**Primary cause of death, n (%)**				
Respiratory failure	-	5	13	14
Cardiovascular	19	-	-	-
Liver diseases	4	-	-	-
Refractory Sepsis/MOF	-	6	2	-
Colon adenocarcinoma	1	-	-	-
Ischemia bowel	1	-	-	-
Intramedullary neoplasia	1	-	-	-
Gastrointestinal bleeding	2	3	-	-

DAD: Diffuse alveolar damage; MOF: multiple organ failure.

### Immunolocalization

**Fig 2:**
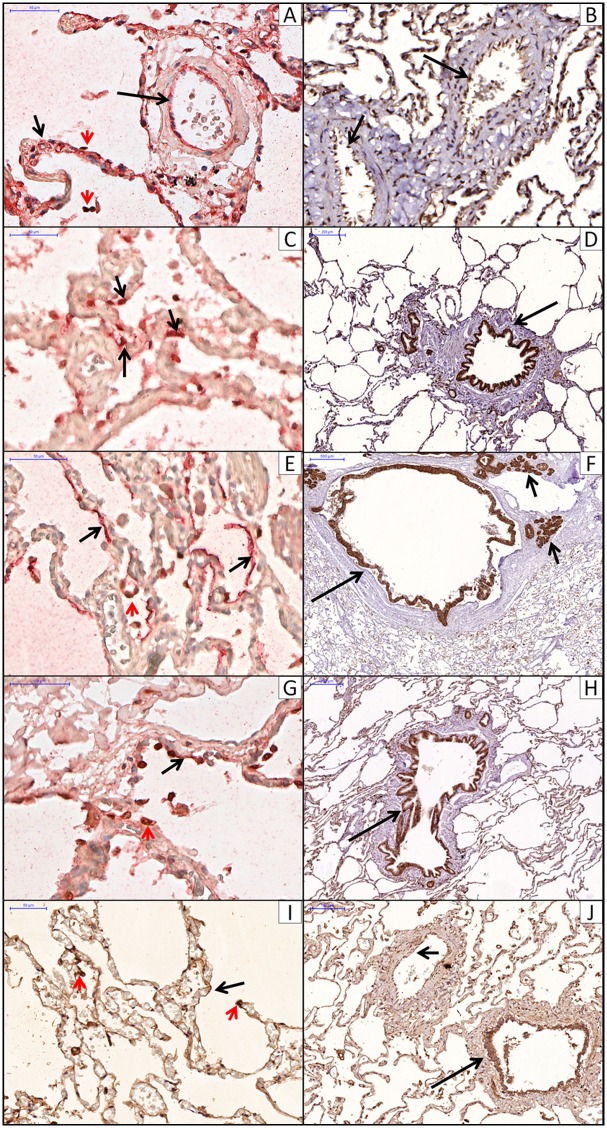
Immunohistochemistry localization of aquaporins, Na-K-ATPase channel and ENaC channel. A: AQP1 in alveolar (short arrow) and arteriolar (long arrow) endothelial cells. Double staining for AQP1 (red) and PII cels (TTF-1, brown, red arrowhead). B: AQP1 in vascular endothelial cells (arrow). C: AQP3 in PII cells (arrows). Double staining for AQP3 (red) and PII cels (TTF1, brown). D: AQP3 in epithelial bronchiolar cells (arrow). E: AQP5 in PI cells lining the alveolar septum (arrow). Double staining for AQP5 (red) and PII cels (TTF1, brown, red arrowhead). F: AQP5 in epithelial bronchiolar cells (long arrow) and submucosal glands (short arrow). G: Na-K-ATPase channel in PI (arrow) and PII (red arrowhead) cells. Double staining for Na-K-ATPase channel (red) and PII cels (TTF1, brown). H: ENaC channel in PI cells lining the alveolar septum (arrow) and PII cells (red arrowhead). I: ENaC channel in epithelial bronchiolar cells (long arrow) and vascular endotelial cells (short arrow).

The alveolar septum presented positive staining in endothelial cells (AQP1), PI (AQP5, ENaC and Na-K-ATPase) and PII (AQP3, ENaC and Na-K-ATPase). The airway respiratory epithelium presented positive staining for all markers except AQP1. The airway submucosal glands presented positive staining for AQP3, AQP5 and Na-K-ATPase. Additionally, ENaC and AQP1 were expressed in the vascular endothelial cells. Channels localization with immunohistochemistry is shown in Fig 2.

### Image Analysis

A mean of 30.7 ± 10.9 mm of alveolar septum was analyzed for each variable for each patient. There was no difference between the total septum length analyzed in the DAD and control groups (30.7 ± 11.2 mm and 30.7 ± 11 mm).

**Fig 3:**
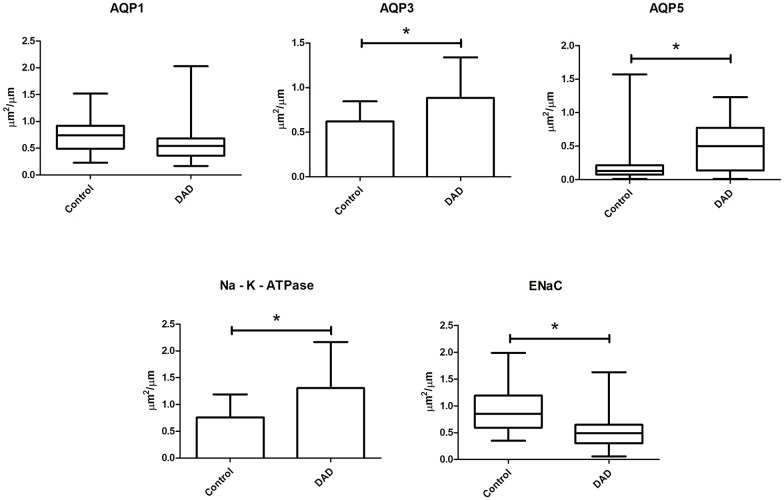
Aquaporin and ion channels expression in the alveolar septum of control and DAD groups.

**Fig 4:**
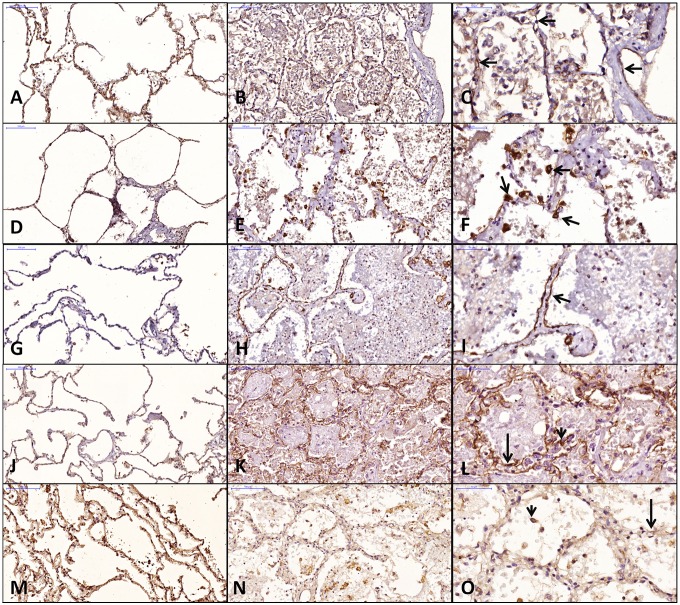
Representative histological images of aquaporins and ion channels expression in DAD and control groups. A, D, G, J and M: control; B, E, H, K and N: DAD; C, F, I, L and O: DAD, higher magnification. AQP 1: A, B, C; AQP 3: D, E, F; AQP 5: G, H, I; Na-K-ATPase: J, K, L; ENaC: M, N, O. C: AQP1 in endothelial cells (arrows); F: AQP3 in PII cels (arrows); I: AQP5 in PI cells (arrow); L: Na-K-ATPase in PI cell (long arrow) and PII cell (short arrow); O: ENaC in PI cell (long arrow) and PII cell (short arrow).

Figs 3 and 4 show the image analysis data for the DAD and control groups. The DAD group showed significantly increased expression of AQP3, AQP5 and Na-K-ATPase and significantly decreased expression of ENaC compared to the control group. AQP1 expression was lower in the DAD than the control group but was only marginally significant (p = 0.07).

Table 3 shows the image analysis data within the DAD group and in controls. The sepsis group accounted for the main differences between DAD and control groups. However, there was no significant difference in protein expression when the subgroups of DAD with different etiologies were compared.

**Table 3:** Protein expression in DAD subgroups and control group.

	Sepsis	H1N1	Lepto	*control*	*p*
AQP1 (μm^2^/ μm)	0.41 [0.39]	0.60 [0.39]	0.57 [0.41]	0.73 [0.48]	NS
AQP3 (μm^2^/ μm)	1.06 ± 0.41	0.7 ± 0.38	0.85 ± 0.5	0.61 ± 0.22	0.011*
AQP5 (μm^2^/ μm)	0.81 [0.46]	0.42 [0.47]	0.18 [0.92]	0.13 [0.12]	0.001*
Na-K-ATPase (μm^2^/ μm)	1.73 [1.04]	1.58 [1.1]	0.48 [2]	0.69 [0.65]	0.004*
ENaC (μm^2^/ μm)	0.53 [0.24]	0.46 [0.58]	0.62 [0.55]	0.83 [0.61]	0.002**

Data are expressed as median [IQR] or mean ± (SD). DAD: Diffuse alveolar Damage, AQP: Aquaporin, ENaC: Epithelial Na^+^ Channel; Lepto: leptospirosis, NS: non-significant.

*, sepsis > control;

**, H1N1 < control.

There was no significant difference in protein expression when only the subgroups of DAD with different etiologies were compared.

## Discussion

In the present study, we found that the expression of water and ion channel proteins in the alveolar septum are altered in patients with acute respiratory failure due to DAD, with an increase in AQP3, AQP5 and Na-K-ATPase and a decrease in ENaC compared to control subjects. However, there was no difference in the expression of the analyzed proteins among DAD groups of different etiologies.

Under normal conditions, transepithelial fluid movement in the alveolar region is mainly driven by active salt transport [6]. Previous experimental studies showed that ENaC and Na-K-ATPase proteins are decreased in acute lung injury/ARDS cell culture and animal models [6, 13, 21, 22]. Furthermore, alveolar fluid clearance is impaired in ARDS patients [9]. Possible mechanisms involved in the active salt transport deficit are epithelial cell death and inflammation, as well as cell junction disruption [5]. In the present study, we showed for the first time that the expression of these proteins is altered in human lung tissue from patients with ARF due to DAD.

Previous experimental studies reported the decreased expression of ENaC and Na-K-ATPase after ALI [5] but, surprisingly, the level of Na-K-ATPase expression was higher in our DAD group compared to control subjects. One possible explanation for the increased expression of Na-K-ATPase in human DAD lungs is the proliferation of PII cells after the acute injury. It was shown in experimental models that ENaC mRNA expression in PI is 3.6 times higher than in PII, whereas the expression of NA-K-ATPase is similar in both types of epithelial alveolar cells [23]. Type I alveolar pneumocytes line over 90% of the alveolar surface and are the most affected cell type during acute lung injury. The immediate physiological response to acute injury is PII proliferation and alveolar septa reepithelization [8]. It is possible that the observed increased expression of Na-K-ATPase in human DAD is due to the increased number of proliferated PII.

The AQPs are a major pathway for osmotically driven water movement across epithelial and microvascular barriers in the lung. However, studies with double-knockout mice for AQP1 and 5 revealed that the absence of these channels did not alter alveolar fluid clearance [5, 24], suggesting that aquaporins add little fluid clearance management if active salt transport is intact. Nevertheless, subsequent studies showed that the expression of lung AQPs are altered after ALI models [10–12, 25, 26]. Although marginally significant, our results showed that the AQP1 level was lower in the DAD group as reported in previous animal models. We also found the increased expression of AQP5 in the DAD groups. The role of AQP5 in edema reabsorption in ALI models is controversial [10–12, 25, 26]. The AQP5 response to lung injury may fluctuate over time and is dependent on the intensity and type of injury [11, 12, 26]. Viral infection and LPS decrease the AQP5 levels [12], whereas bleomycin inhalation and thoracic irradiation lead to the increased expression of AQP5 [11, 26]. Similar to our findings, a previous report on patients with leptospirosis [25] showed an increased number of aquaporin 5 positive cells within the alveolar tissue, consistent with the hypothesis that osmotically driven water may play a role as a compensatory effect when salt transport is impaired.

AQP3 is an aquaglycerolporin expressed at the basolateral membrane of type II pneumocytes [27]. Not only important for alveolar fluid clearance management, glycerol transport is important for cell regeneration and tumor progression because glycerol may be a fuel for cell metabolism by activating MAP kinase to stimulate cell proliferation [3]. Therefore, the increased expression of AQP3 in our DAD group supports a role for AQP3 in tissue reepithelization and regeneration and may also be related to PII cell proliferation.

We did not find any significant difference in the expression of water and ion channels among DAD with different etiologies (sepsis, H1N1 virus and leptospirosis). This finding suggests that the impairment of transport channels in DAD is mainly dependent on the extension or intensity of lung injury than on its primary pathophysiology.

One limitation of our study was the limited access to clinical data at the time of death because some patients died in other institutions and were referred for autopsy. Mechanical ventilation and clinical management, such as volume expansion and fluid management, could interfere with our results. However, these procedures are commonly used as a rescue save/lifesaving therapy and their effects cannot be separated or ruled out under real world conditions. Finally, the absence of differences reported within the DAD group with different etiologies may be relate to the severity of cases, and we cannot assume that in less severe cases or during the initial stage of the syndrome that the expression of ion and water transport channels is similar.

In conclusion, we showed that water and ion channels are altered in patients with acute respiratory failure due to DAD, with an increase in AQP3, AQP5 and Na-K-ATPase expression and a decrease in ENaC expression. The cause of DAD does not seem to influence the level of impairment of these channels.

## Supporting Information

S1 Table Individual data of protein content obtained with image analysis. (XLS)
